# In Vitro Culture of Single Bovine Embryos with Microwell Plates Made of Poly(dimethylsiloxane) Cured under Low Pressure

**DOI:** 10.1155/2018/7546986

**Published:** 2018-06-06

**Authors:** Daisaku Iwamoto, Nobuhiro Kato, Shunji Taniguchi, Yoshitomo Taguchi, Masao Kishi, Kazuhiro Saeki

**Affiliations:** ^1^Faculty of Biology-Oriented Science and Technology, Kindai University, Kinokawa, Wakayama 649-6493, Japan; ^2^Wakayama Prefecture Livestock Station, 1 Mirozu Susami, Wakayam, Japan; ^3^University Farm, Kindai University, Aridagawa, Wakayama 643-0531, Japan

## Abstract

Single embryo culture is useful for assessing the developmental competence of an embryo in detail. Recently, a device made of poly(dimethylsiloxane) (PDMS), which is biocompatible and nontoxic, has been widely used for culture various types of cells. However, PDMS plates are porous, causing the serious osmolality increment of the medium (over 600 mOsm/kg from Day 4 to Day 7). Here, we report that curing the PDMS under low pressure (LP-PDMS) greatly reduced the porosity, resulting in a constant osmolality of the medium. The blastocyst rate of single bovine embryos cultured with LP-PDMS microwell (MW) plates was the same as that of group-cultured embryos (25 embryos/50 *μ*l droplet; control, P>0.05). These results indicate that MWs on a plate made of PDMS cured under low pressure can be successfully used for individual embryo culture.

## 1. Introduction

Methods for culturing bovine embryos have been improving over the past 30 years. Mammalian embryos cultured in a group culture system (25-200 embryos per 50-400 *μ*l of a medium droplets) have a higher developmental ability than embryos cultured individually or in small groups [1–4]. However, it is difficult to culture large groups of embryos, because the number of oocytes collected by transvaginal ovum pick-up is limited and depends heavily on the physiological and reproductive states of donor animals. Culturing mammalian embryos individually makes it possible to observe each embryo in detail and continuously. It has been reported that embryos with high pregnancy rates can be selected with the assessments during early development of single embryos using time-lapse cinematography [5]. Therefore, there is a need for culture systems that can culture embryos individually or in small groups.

Vajta et al. (2000) reported that bovine embryos can be cultured to the blastocyst stage individually in microwells (MWs) made on the bottom of a plastic culture dish with a darning needle [well of the well (WOW) system]. However, the MWs have some disadvantages for culturing embryos; the wells are not uniform due to their fabrication by hand, and toxic materials may be generated during melting the plastic dishes using a heated needle [6].

Poly(dimethylsiloxane)(PDMS), a type of silicon, which is commonly used as a material of contact lenses, is optically transparent and biocompatible and can be manufactured straightforwardly by rapid prototyping [7]. However, PDMS is so porous that the osmolality of culture media is increased by absorption of water into the PDMS [8], making it difficult to use the PDMS for culture of cells and embryos. The porosity of the PDMS is due to hydrogen generated during the curing reaction of PDMS. Therefore, we hypothesized that the porosity might be decreased by curing PDMS under low pressure to remove the hydrogen from PDMS.

In this study, we analyzed the characteristics of the PDMS cured under low pressure and examined whether bovine embryos could develop to blastocyst in a MW plate made of PDMS.

## 2. Materials and Methods

### 2.1. Fabrication of PDMS Microwells (PDMS MW) Plates Cured under Low Pressure

A PDMS MW plate was fabricated by using soft lithography as reported previously [9]. Poly(dimethylsiloxane) was prepared by casting prepolymer (SYLGARD® 184 silicone elastomer base curing agent, Dow Corning Corp., Midland, MI, USA) at a 1:10 curing agent-to-base ratio against positive relief features. The relief features were composed of SU-8 and fabricated on a thin glass wafer by using backside diffused-light photolithography. PDMS MW plates were cured at 60°C under atmospheric (regular PDMS) or low pressure (LP-PDMS) with a vacuum desiccator (-0.08MPa), respectively.

### 2.2. Imaging Surface of PDMS Plates by an Atomic Force Microscope (AFM)

The surface structures of regular and LP-PDMS plates were observed with an AFM (SPI 3800, Seiko Instruments Inc., Chiba, Japan) using the dynamic force (tapping) mode. It took approximately 20 minutes to scan a single area. Scanned images stored in the computer, which controls the AFM, were analyzed for fine surface structure using AFM software.

### 2.3. Measurement of Osmolality of Culture Medium

A vapor pressure osmometer (Vapro 5520, Wescor Inc. Logan, UT, USA) was used to measure osmolality of a culture medium [modified synthetic oviduct fluid medium; mSOFM [10]]. Regular and LP-PDMS plates (10mm×10mm) with a hole (1mm diameter) punctured by biopsy punch (Kai Corp, Japan) were attached to a 35-mm cell culture dish and incubated in 2 mL in mSOFM covered with 2 mL paraffin oil at 39°C in 5% CO_2_, 5% O_2_, and 90% N_2_ with high humidity. Two mL medium without a PDMS plate covered with 2 mL paraffin oil was used as a control. The osmolality of mSOFM in the well was measured at 24 h intervals over a 168-hour period. To measure osmolality, 10 *μ*l of culture medium was taken from the well of PDMS and placed on the sample loading area in the osmometer.

### 2.4. Production of Bovine Embryos Fertilized In Vitro

Bovine oocytes were matured as reported previously [10]. Briefly, bovine ovaries were obtained from a local slaughterhouse and were transported in saline at 20-25°C. Cumulus-oocyte complexes (COCs) were collected from the ovaries and washed with 25 mM Hepes-buffered TCM199 with Hanks' salts (199H: Gibco, Invitrogen Life Technologies, Tokyo, Japan) supplemented with 5 % (v/v) FBS and 25 *μ*l/ml gentamicin (FBS199H). The washed COCs were matured for 18-21 hours in 50 *μ*l of 25 mM Hepes-buffered TCM199 with Earle's salts (199E: Gibco) supplemented with 5% FBS, 0.5 mM sodium pyruvate, 25 *μ*g/ml gentamicin, 0.02 AU/ml FSH (Antrin: Kyoritsu Pharmaceutical, Tokyo, Japan), and 1 *μ*g/ml estradiol-17*β* covered with paraffin oil at 39°C in 5% CO_2_ in air in high humidity (10 COCs/droplet).

*In vitro* fertilization (IVF) was carried out as described previously [10]. Briefly, frozen-thawed spermatozoa were washed with a discontinuous gradient Percoll solution (Amersham Biosciences, Uppsala, Sweden) [11]. Matured COCs cultured for 21 hours in the maturation medium were inseminated with Percoll washed spermatozoa in a defined medium [12] modified by excluding glucose and supplemented with heparin (yielding final concentrations of 2 x 10^6^ sperm and 10 *μ*g heparin/ml). Oocytes and spermatozoa were coincubated for 18 h under 5% CO_2_ in air at 39°C with high humidity (10 COCs/100 *μ*l). Following IVF, the surrounded cumulus cells and spermatozoa were removed from the oocytes and the oocytes were cultured in LP-PDMS MWs individually with mSOFM covered with paraffin oil at 39°C in 5% CO_2_, 5% O_2_, and 90% N_2_ with high humidity. For a control, the oocytes were cultured in a 50-*μ*l mSOFM droplet covered with paraffin oil at 39°C in 5% CO_2_, 5% O_2_, and 90% N_2_ with high humidity. Rates of cleavage and blastocysts were examined 168 hours after insemination under stereomicroscope (×60).

### 2.5. Differential Staining of ICM and TE Cells in Blastocysts

To examine the quality of blastocysts, their inner cell mass (ICM) and trophectoderm (TE) cells were separately stained as described previously [13]. Briefly, blastocysts were incubated in the first solution [TCM199 containing 1% Triton X100 and 100 *μ*g/ml propidium iodide (PI)] for 30 seconds and then incubated in the second solution [fixative solution: PBS containing 0.1% polyvinyl alcohol (PVA), 4% paraformaldehyde and 25 *μ*g/ml Hoechst 33342] for 30 min at room temperature. The fixed and stained blastocysts were washed twice in PBS containing PVA, mounted on a drop of Vectashield (Vector Laboratories, Burlingame, CA, USA), and observed under a fluorescence inverted microscope (IX-71; Olympus, Japan). The numbers of total and TE cells were counted from Hoechst (blue) and PI (red) image, respectively. The number of ICM cells was evaluated by subtracting the number of TE cells from the number of total cells.

### 2.6. Experimental Designs

#### 2.6.1. Experiment 1: Characterization of PDMS Cured under Low Pressure

To characterize LP-PDMS, first the surface structure of a LP-PDMS plate was observed using AFM. In applying the PDMS plate to the culture of bovine embryos, we then measured the osmolality of the medium in regular and LP-PDMS plates every 24 hours to investigate whether the difference in the surface structure affects the osmolality of the medium.

#### 2.6.2. Experiment 2: Early Development of Bovine IVF Embryos

**Figure 1:**
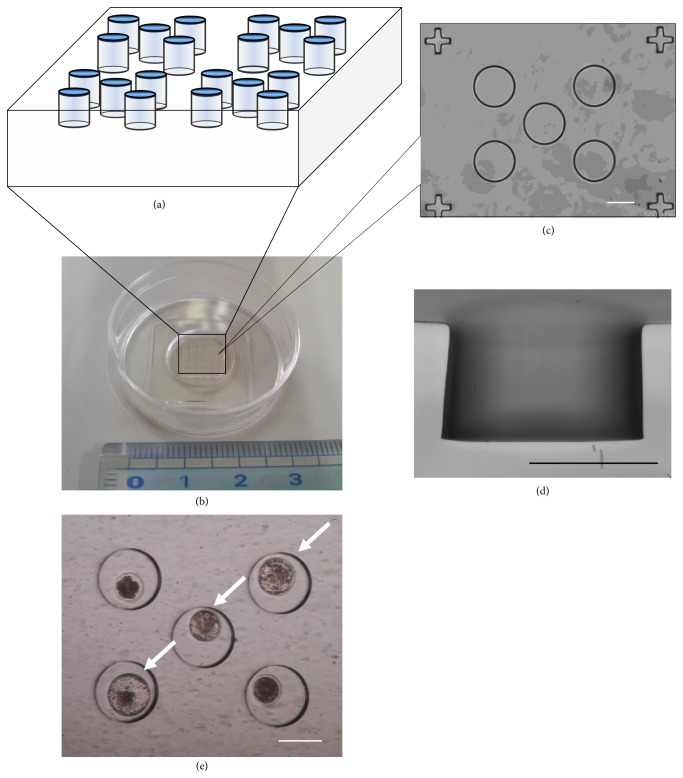
**Images of a PDMS MW plate attached on the bottom of a glass-bottom dish.** (a) Schematic diagram showing a PDMS MW plate. Some cylindrical MWs were fabricated on the plate surface. (b) Poly(dimethylsiloxane) microwell (PDMS MW) plate was attached to the bottom of a glass-bottom dish by oxygen plasma treatment. (c) Transmission and (d) median section images of the PDMS MW. The cylindrical MW was 300 *μ*m in diameter and 200 *μ*m in depth. (e) One bovine embryo was introduced into each PDMS MW. Blastocysts (arrow) were observed in the PDMS MW after 7 days of culture. Scale bar: 200 *μ*m.

We examined whether a LP-PDMS MW plate can be used for single embryo culture. After IVF, 375 fertilized bovine embryos were divided into five different culture systems. One embryo or 25 embryos were cultured in a 50 *μ*l droplet of culture medium (single embryo culture or group culture, respectively) as a control [4]. For WOW, the MWs were made by pressing a darning needle (BLS, Budapest, Hungary) on the bottom of culture dish in 50 *μ*l droplet, and then each embryo was transferred individually into each MW. Regular and LP-PDMS MW plates were attached on glass-bottom dish by plasma oxygen treatment and used for embryo culture. Each MW was of cylindrical shape (Figure 1, 300 *μ*m diameter and 200 *μ*m depth). Each embryo was transferred individually into each MW covered with 2 ml mSOFM. These embryos were cultured for 168 h at 39°C in 5% CO_2_, 5% O_2_, and 90% N_2_ with high humidity.

### 2.7. Statistical Analysis

All experiments were replicated at least three times. Data were analyzed using Stat View software (Abacus Concepts, Stat View, version J-4.11, Abacus Concepts, Berkeley, CA, USA). The data obtained from values of osmolality and cell numbers of the blastocysts were analyzed with Tukey-Kramer post hoc tests for multiple comparisons following ANOVA. Differences of P<0.05 were considered to be significant. The data of cleavage and blastocyst rates were analyzed with Fisher's protected least significant difference (PLSD) tests following ANOVA. Differences of P<0.05 were considered to be significant.

## 3. Results

### 3.1. Experiment 1: Effects of Low Pressure Curing on Porosity of PDMS Plate

**Figure 2:**
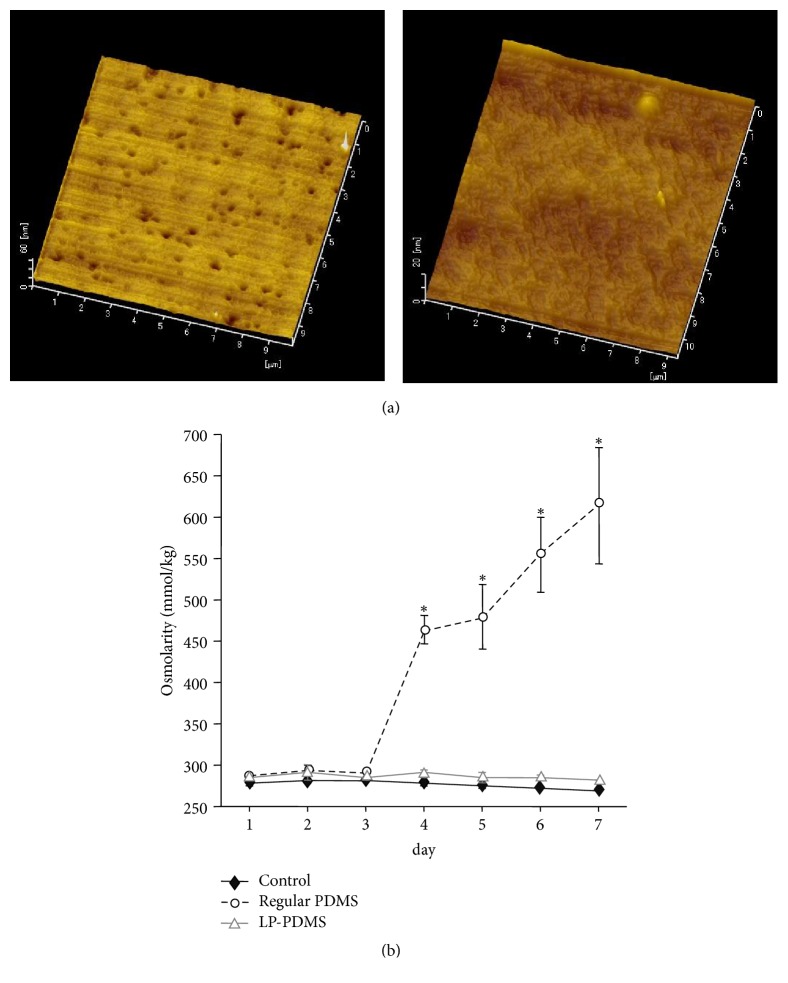
**Characteristics of PDMS plate cured under low pressure.** (a) AFM images of the surfaces of regular and LP-PDMS plates. (Left) Regular PDMS plate cured under atmospheric pressure. (Right) LP-PDMS plate cured under low pressure (-0.08 MPa). Scale bar: 1 *μ*m. (b) Effect of PDMS curing conditions on osmolality of culture medium in regular and LP-PDMS MW. Culture medium added to plastic dish was used as a control. Asterisks were denoted significant differences between regular PDMS MW group (open circle) and other groups (LP-PDMS MW group; open triangle and control; closed diamond) in each Day (P<0.05).

As shown by AFM images, the regular PDMS had many pores whereas the LP-PDMS had no pores (Figure 2(a)). From Day 4 to Day 7, the osmolality of the medium doubled in the regular PDMS plate (from 290 to 610 mOsm/kg) while it stayed constant in the LP-PDMS plate and control (without PDMS) (Figure 2(b)).

### 3.2. Experiment 2: In Vitro Culture of Single Bovine Embryos with a LP-PDMS MW Plate

The cleavage rates of embryos cultured in different systems (group culture, single culture, WOW, regular PDMS MW, and LP-PDMS MW) ranged from 65% to 75% and were not significantly different (P>0.05) (Table 1). The blastocyst rate with LP-PDMS was similar to those of the group culture (control) and WOW (32% versus 38% and 15%, respectively; P>0.05). On the other hand, the rate with a regular PDMS MWs (6%) was lower than those of the group culture, WOW, and LP-PDMS MWs (P<0.05). The cell numbers of ICM and TE of the embryos were not significantly different among these groups (Table 2; P>0.05).

## 4. Discussion

In the present study, we demonstrated that the surface of LP-PDMS was nonporous and the osmolality of a culture medium with LP-PDMS MW plates did not increase. Poly(dimethylsiloxane) has a porous feature because of the generation of hydrogen gas during curing of the PDMS prepolymer. Heo et al. (2007) showed that parylene coating of microfluidic systems made of PDMS, which made the coating pinhole free, suppressed the increase of osmolality of the culture medium, and significantly improved blastocyst development of mouse embryos compared to the identical system with no parylene [8]. The pores on the plate made of PDMS appear to account for the increase of osmolality of the culture medium. Our results suggest that few hydrogen bubbles form in the PDMS when the plates are cured under low pressure. The osmolality of medium in plates made of regular PDMS did not increase until Day 3, but, however, increased significantly on Day 4. The reason of the delay in increasing the osmolality is not clear, but the delay might suggest that the pores on the plates made of regular PDMS would be so fine that it might take time for the medium in the plates to permeate into the numerous fine pores on the plate.

With such MW plates, bovine embryos can be cultured individually. The blastocyst rate of bovine embryos cultured in LP-PDMS MWs was similar to that of group culture (25 embryos/50 *μ*l droplet, P>0.05). On the other hand, the blastocyst rate of bovine embryos cultured in regular PDMS MWs was low and similar to that of single embryo culture and significantly lower than those of other groups (P<0.05). Early embryos are more sensitive to the culture environment than somatic cells [14]. Increased osmolality of culture media adversely affects the early development of mammalian embryos [15–18]. The present results suggest that the increase of osmolality in regular PDMS is detrimental to the in vitro development of bovine embryos.

In our study, the blastocyst rate with WOW was lower than that with group culture (P<0.05). Although some studies have shown that mammalian embryos can be cultured individually into blastocysts in MWs, the shape and volume of the MW were different in each study [5, 6, 19, 20]. The shape of the WOWs was not uniform due to their manual fabrication [6, 21], which might decrease blastocyst rate of bovine embryos. Further studies are needed to determine whether the shape and volume of MWs influence in vitro development of mammalian embryos.

In conclusion, we found that PDMS cured under low pressure was nonporous and that osmolality of culture medium was not increased in MWs made of the PDMS. We demonstrated that bovine embryos can be individually cultured in MWs made of the PDMS plate.

## Figures and Tables

**Table 1:** In vitro development of bovine IVF embryos cultured with PDMS MWs^1^.

Culture system	No of embryos cultured	No (%) of embryos cleaved^2^	No (%) of blastocysts^3^
Group culture (control; 25 embryos/50*μ*l droplet)	75	49 (65)	19 (38)^a^
Single embryo culture (1 embryo/50*μ*l droplet)	75	53 (71)	0 (0)^c^
WOW (1 embryo/1 well)	75	50 (67)	11 (22)^b^
Regular-PDMS MW (1 embryo/ 1 well)	75	53 (71)	3 (6)^c^
LP-PDMS MW (1 embryo/1 well)	75	56 (75)	18 (32)^a,b^

^1^The experiments were replicated three times.

^2^The percentage of cultured embryos.

^3^The percentage of cleaved embryos.

^a-c^Superscripts in the column indicate significant difference.

**Table 2:** Cell number of blastocysts cultured with PDMS MWs.

Culture system	No of blastocyst	No of cells			% of ICM/Total
		Total	ICM	TE	
Group culture (control)	19	98±5	31±3	66±4	32
WOW	9	86±9	29±3	56±6	34
Regular PDMS	3	110±12	40±8	70±6	36
LP-PDMS	17	103±6	34±3	68±5	33

## Data Availability

The data used to support the findings of this study are available from the corresponding author upon request.
